# Checklist of ascidians (Chordata, Tunicata) from the southern Gulf of Mexico

**DOI:** 10.3897/zookeys.832.31712

**Published:** 2019-03-19

**Authors:** Lilian A. Palomino-Alvarez, Rosana Moreira Rocha, Nuno Simões

**Affiliations:** 1 Unidad Multidisciplinaria de Docencia e Investigación Sisal (UMDI-SISAL), Facultad de Ciencias, Universidad Nacional Autónoma de México, Puerto de abrigo s/n, Sisal, CP 97356 Yucatán, Mexico; 2 Zoology Department, Universidade Federal do Paraná – UFPR, CP 19020, CEP 81531-980, Curitiba, PR, Brazil; 3 Laboratorio Nacional de Resiliencia Costera Laboratorios Nacionales, CONACYT, Mexico City, Mexico; 4 International Chair for Coastal and Marine Studies, Harte Research Institute for Gulf of Mexico Studies, Texas A&M University, Corpus Christi, Texas, USA

**Keywords:** Ascidiacea, biodiversity, Gulf of Mexico, Yucatán

## Abstract

This study is the first inventory of ascidians from shallow waters (0–25 m) of coastal and reef habitats in the southern Gulf of Mexico where ascidian diversity is poorly known. Sampled environments in 14 locations (38 sites) with 134 samples collected from 2015 to 2017 included coral reefs, coastal lagoons, mangroves, seagrass, ports, and artificial platforms. The 31 identified species comprise 19 genera and 13 families. Ten species are newly reported in the Gulf of Mexico: *Ascidiapanamensis* Bonnet & Rocha, 2011; *Ecteinascidiastyeloides* (Traustedt, 1882); *Cystodytesroseolus* Hartmeyer, 1912; Eudistomaaff.amanitum Paiva & Rocha, 2018; *Eudistomarecifense* Millar, 1977; *Euherdmaniafasciculata* Monniot, 1983; Euherdmaniaaff.vitrea Millar, 1961; *Polycarpacartilaginea* (Sluiter, 1885); *Botrylloidesmagnicoecum* (Hartmeyer, 1912) and *Didemnumgranulatum* Tokioka, 1954. Two new species will be described separately (*Clavelina* sp. and *Pyura* sp.). This study provides the first records for 26 species ascidians for the region as well as describes increased distributions of ten Atlantic species. Thus, our data provide a starting point for future ecological, experimental and taxonomic studies of ascidians of the Gulf of Mexico.

## Introduction

The Ascidiacea is the most diverse class of tunicates with ca 3000 recognized species, with representatives found in all marine habitats (Shenkar and Swalla 2011). Local ascidian species diversity depends primarily on availability and diversity of hard substrates, as well as temperature and salinity (Lambert 2005), while population density depends on food availability (organic particles suspended in water; Monniot et al. 1991). Ascidians are active suspension filter-feeders and are key organisms at times when they contribute to the control of phytoplankton (Petersen and Riisgard 1992) and may reduce eutrophication or contaminant concentration (Naranjo et al. 1996, Draughon et al. 2010). Many species colonize most artificial substrates and thereby become among the dominant members of “fouling” communities (Carballo 2000). Among foulers there are species known for their invasion potential worldwide (Lambert 2005). Ascidians are also known because of the presence of bioactive metabolites with potential biomedical interest (Erba et al. 2001).

Ascidian diversity in the Gulf of Mexico includes records of 79 species in 15 families in the northern Gulf of Mexico (Carballo 2000, Cole and Lambert 2009, CONABIO 2016, Fortaleza and Lotufo 2018). The southern Gulf of Mexico, however, is much less known and, despite the ecological and biotechnological importance of ascidians, only includes nine reported species: *Aplidiumexile* (Van Name, 1902); *Polyclinumconstellatum* Savigny, 1816; *Ecteinascidiaturbinata* Herdman, 1880; *Eudistomacapsulatum* (Van Name, 1902); *Eudistomahepaticum* (Van Name, 1921); *Stomozoaroseola* (Millar, 1955); *Botrylloidesniger* Herdman, 1886; *Symplegmabrakenhielmi* (Michaelsen, 1904) and *Symplegmaviride* Herdman, 1886 (Van Name 1945; Carballo 2000). Essentially there are no studies from Mexico and the seven reported species is far below the expected number considering the great diversity of suitable habitats. To fill this gap, here we provide an inventory of the coastal species of ascidians in reefs and other shallow habitats in the southern Gulf of Mexico, along the Yucatán Peninsula.

## Material and methods

**Figure 1.**
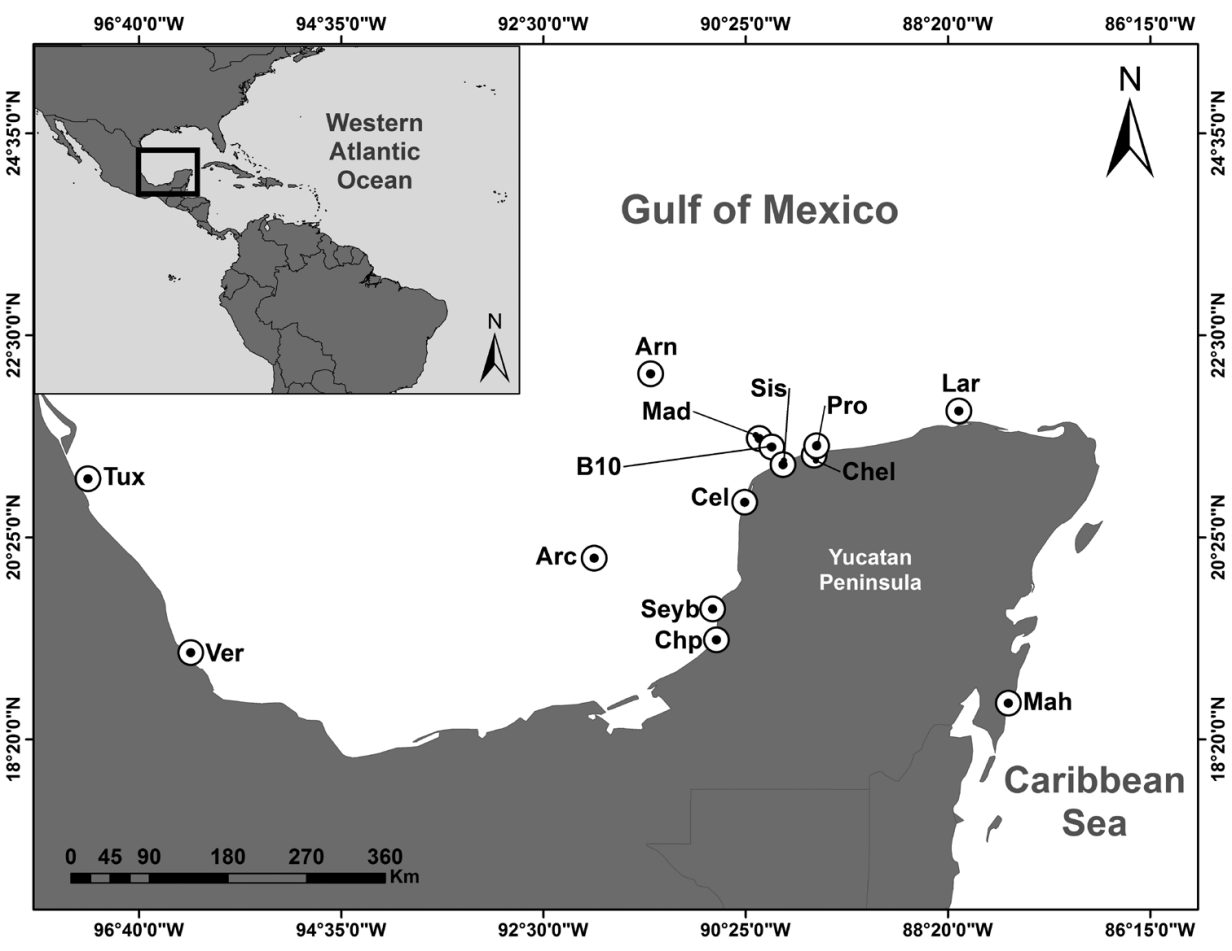
Study area in the southern Gulf of México. Abbreviations: Tuxpan Reef – Tux, Veracruz Reef – VeR, Arcas Cay Reef – Arc, Seybaplaya – Sey, Champotón – Chp, Celestún – Cel, Arenas Cays – Arn, Madagascar Reef – Mad, Bajo 10 Reef – B10, Chelém Coastal Lagoon – Chel, Progreso Harbor – Pro, Ría Lagartos – Lag, Mahahual Harbor – Mah, and Sisal Harbor – Sis.

Samples were collected in 14 locations and 38 sites from 2015 to 2017 in coral reefs, coastal lagoons, mangroves, seagrass, ports and artificial platforms by free diving and SCUBA, in the states of Veracruz, Tabasco, Campeche, Yucatan and Quintana Roo (Fig. 1, Table 2). Specimens were anesthetized in menthol and fixed with 4% formaldehyde in seawater. External characters of ascidians provide little information for determining their taxonomy and therefore dissection is required, for which a list of multiple characters is available (Monniot and Monniot 1972; Monniot et al. 1991; Rocha et al. 2012). Dissection was carried out following Monniot and Monniot (1972) and internal structures were stained with Harris hematoxylin dye (see: https://bocasarts.weebly.com/tunicate-tools.html). Families and genera were identified following Rocha et al. (2012) for species of the Atlantic Ocean.

Specimens were deposited in the Colección de Ascidias del Golfo de México (CAGoM), which is part of the collection of the Marine Invertebrates of Gulf of Mexico, National Autonomous University of Mexico (UNAM), Mérida, Yucatán. The resulting dataset has been uploaded to the Zenodo data repository (Alvarez et al. 2018).

## Results

In 134 samples we identified 31 species in 19 genera and 13 families in 14 locations at 38 sites (see Table 2). We report ten species for the first time in the Gulf of Mexico: *Ascidiapanamensis* Bonnet & Rocha, 2011; *Cystodytesroseolus* Hartmeyer, 1912; *Ecteinascidiastyeloides* (Traustedt, 1882); Eudistomaaff.amanitum Paiva & Rocha, 2018; *Eudistomarecifense* Millar, 1977; *Euherdmaniafasciculata* Monniot, 1983; Euherdmaniaaff.vitrea Millar, 1961; *Polycarpacartilaginea* (Sluiter, 1885); *Botrylloidesmagnicoecus* (Hartmeyer, 1912) and *Didemnumgranulatum* Tokioka, 1954 (Table 1) and two new species that will be reported somewhere else. Most specimens were found on natural substrates (rocks, corals and algae) followed by artificial substrates (oil platforms, docks and sunken ships).

**Table 1.** Species checklist of ascidians in south Gulf of Mexico. Abbreviations: (Tux) Reef Tuxpan, (VeR) Veracruz Reef, (Arc) Reef Arcas Cay, (Sey) Seybaplaya, (Chp) Champotón, (Cel) Celestún, (Arn) Arenas Cays, (Mad) Reef Madagascar, (B10) Reef Bajo 10, (Chel) Coastal Lagoon Chelém, (Pro) Progreso Harbor, (Lar) Ría Lagartos (Sis) Sisal Harbor, and (Mah) Mahahual Harbor. (*) New records for Gulf of Mexico.

	Tux	Ver	Arc	Sey	Chp	Cel	Arn	Mad	B10	Chel	Pro	Lar	Mah	Sis	
# spp	4	2	8	3	2	2	4	15	10	6	12	7	1	2	# Sites
**Order Phlebobranchia**															
** Ascidiidae **															
Ascidiapanamensis*			•					•							2
* Phallusia nigra *	•	•			•					•	•				4
** Corellidae **															
* Corella minuta *			•												1
** Perophoridae **															
Ecteinascidiastyeloides*			•						•	•		•			4
* Ecteinascidia turbinata *	•		•				•			•	•				4
**Order Aplousobranchia**															
** Clavelinidae **															
* Clavelina oblonga *				•				•							2
*Clavelina* sp.							•								1
** Polycitoridae **															
* Cystodytes dellechiajei *									•						1
Cystodytesroseolus*								•	•		•				3
Eudistomaaff.amanitum*								•	•			•			3
* Eudistoma clarum *								•			•	•			3
* Eudistoma hepaticum *				•		•		•	•		•				5
* Eudistoma obscuratum *								•				•			2
* Eudistoma olivaceum *					•			•		•	•		•		5
Eudistomarecifense*								•	•		•				3
** Stomozoidae **															
* Stomozoa roseola *	•														
** Holozoidae **															
* Distaplia bermudensis *								•							1
** Didemnidae **															
* Polysyncraton amethysteum *								•			•				2
* Lissoclinum fragile *												•			1
* Didemnum duplicatum *			•	•				•	•		•				5
Didemnumgranulatum*								•				•			2
** Polyclinidae **															
* Polyclinum constellatum *										•					1
** Euherdmaniidae **															
Euherdmaniafasciculata*											•	•			2
Euherdmaniaaff.vitrea*								•	•						2
**Order Stolidobranchia**															
** Styelidae **															
Polycarpacartilaginea*			•				•								2
* Polycarpa spongiabilis *			•				•								2
Botrylloidesmagnicoecus*								•							1
* Botrylloides niger *	•	•	•			•				•					3
** Pyuridae **															
*Pyura* sp. 1									•		•				2
* Microcosmus exasperatus *									•		•			•	2
** Molgulidae **															
* Molgula occidentalis *														•	1

**Table 2.** Study localities in south of Gulf of Mexico.

Localities	Latitude / Longitude
Progreso Harbor	
Pro 1	21°19'56.4"N, 89°41'17.8"W
Pro 2	21°20'58.1"N, 89°40'49.1"W
Pro 3	21°21'41.12"N, 89°41'7.02"W
Reef Arcas Cay	
Arc 1	20°12'11"N, 91°58'56"W
Arc 2	20°12'13"N, 91°58'34"W
Arc 3	20°12'16.62"N, 91°57'48.13"W
Arc 4	20°12'16.9"N, 91°58'39.8"W
Arc 5	20°12'17.17"N, 91°57'48.06"W
Arc 6	20°12'19.95"N, 91°57'39.19"W
Arc 7	20°12'31.1"N, 91°57'51.37"W
Arc 8	20°12'32.14"N, 91°57'41.04"W
Arc 9	20°12'35.6"N, 91°58'0.7"W
Arc 10	20°12'36.36"N, 91°57'51.08"W
Arc 11	20°12'41.6"N, 91°57'49.1"W
Arc 12	20°12'56.6"N, 91°58'31.3"W
Reef Arenas Cays	
Arn 1	22°6'12.73"N, 91°23'41.64"W
Arn 2	22°6'54.11"N, 91°23'42.17"W
Reef Madagascar	
Mad 1	21°26'16.1"N, 90°16'36.6"W
Mad 2	21°26'16.4"N, 90°16'39.3"W
Mad 3	21°26'17.5"N, 90°16'34.9"W
Mad 4	21°26'17.7"N, 90°16'39.7"W
Reef Bajo 10	
B10	21°20'58"N, 90°8'52.3"W
Celestún	
Cel 1	20°46'43.4"N, 90°25'36.1"W
Cel 2	20°49'0.4"N, 90°25'59.3"W
Champotón	
Chp 1	19°21'18.98"N, 90°43'35.77"W
Chp 2	19°21'41.8"N, 90°43'3.4"W
Coastal Lagoon Chelém	
Chel 1	21°15'47"N, 89°44'28.82"W
Chel 2	21°15'55.26"N, 89°42'39.08"W
Mahahual Harbor	
Mha	18°42'30"N, 87°42'40"W
Sisal Harbor	
Sis	21°10'4.29"N, 90°1'55.3"W
Ría Lagartos	
Lar 1	21°43'19.9"N, 88°13'11.8"W
Lar 2	21°43'23.6"N, 88°13'6.5"W
Lar 3	21°43'8.4"N, 88°12'27.1"W
Seybaplaya	
Sey 1	19°39'3.3"N, 90°42'31.4"W
Sey 2	19°40'44.3"N, 90°45'20.6"W
Sey 3	19°44'11.7"N, 90°48'22.8"W
Reef Tuxpan	
Tux	21°1'21.5"N, 97°11'27.4"W
Veracruz Reef	
VeR	19°12'25.5"N, 97°4'7"W

### Systematics

#### Subphylum Tunicata Lamarck, 1816

##### Class Ascidiacea Blainville, 1824

###### Order Phlebobranchia Lahille, 1886

####### Family Ascidiidae Herdman, 1882

######## Genus *Ascidia* Linnaeus, 1767

######### Ascidia panamensis

Taxon classificationAnimaliaPhlebobranchiaAscidiidae

Bonnet & Rocha, 2011

Fig. 2A

########## Material examined.

CAGoM-0023, Mad 1, 9 m, 20-04-2015, leg. L. Palomino-Alvarez; CAGoM-0182, Arc 3, 3 m, 30-10-2015, leg. L. Palomino-Alvarez; CAGoM-0187, CAGoM-00189, Arc 6, 2 m, 30-10-2015, leg. L. Palomino-Alvarez; CAGoM-0190, CAGoM-0191, Arc 8, 2 m, 31-10-2015, leg. L. Palomino-Alvarez.

########## Remarks.

These specimens are of uniformly dark coloration inside the siphons, in contrast to specimens from Panama which have white lines between the siphon lobes (Bonnet and Rocha 2011a). Mexican specimens are also smaller with conical papillae on the tunic in the area of the oral siphon, the shape of the dorsal tubercle is variable, and the anus is smooth. All specimens were found under rocks, two to three specimens per rock, in association with *Polycarpacartilaginea* (Sluiter, 1898) and *Corellaminuta* Traustedt, 1882. Symbiosis was also noted with palaemonid crustaceans (*Ascidoniamiserabilis* (Holthuis, 1951)) living in the pharynx of some of the larger specimens.

########## Global distribution.

Mexico (as described here) and Panamá (Bonnet and Rocha 2011a).

######## Genus *Phallusia* Savigny, 1816

######### Phallusia nigra

Taxon classificationAnimaliaPhlebobranchiaAscidiidae

Savigny, 1816

########## Material examined.

CAGoM-0062, Pro 1, 4 m, 26-05-2015, leg. L. Palomino-Alvarez; CAGoM-0085, CAGoM-0089, Sey 1, 11 m, 12-06-2015, leg. L. Palomino-Alvarez; CAGoM-0733, Sis, 1 m, 21-03-2018, leg. Bryan Flores;

Photographed record (no specimens in the collection): Tux, 5 m, 21-09-2015.

########## Remarks.

This species was only recorded on artificial substrates and shallow rocks near the shore.

########## Global distribution.

United States (Van Name 1921, 1945; Plough 1978), Bermudas (Herdman 1882; Verrill 1900; Van Name 1902, 1945; Monniot 1973), Panama (Collin et al. 2005; Rocha et al. 2005; Bonnet and Rocha 2011a), Curaçao (Millar 1962; Goodbody 1984), Venezuela (Bermudez and Grimaldi 1975), Guadeloupe (Monniot 1983a), Martinique (Monniot 2018a), French Guiana (Monniot 2016), Brazil (Van Name 1921, 1945; Millar 1958; Monniot 1970; Rodrigues 1962; Rocha and Costa 2005; Bonnet and Rocha 2011a; Dias et al. 2012), South Africa (Herdman 1880), Angola (Millar 1965), Greece (Kondilatos et al. 2010), Suez Canal (Harant 1927; Ghobashy and Abdel Messeih 1991), Israel (Pérès 1958; Shenkar 2012), Red Sea (Michaelsen 1918; Savigny 1816), Micronesia (unconfirmed – Nishikawa 1984; Lambert 2003).

####### Family Corellidae Lahille, 1888

######## Genus *Corella* Alder & Hancock, 1870

######### Corella minuta

Taxon classificationAnimaliaPhlebobranchiaCorellidae

Traustedt, 1882

Fig. 2B

########## Material examined.

CAGoM-0369, Arc 5, 2 m, 21-08-2016, leg. L. Palomino-Alvarez; CAGoM-0384, Arcas Cay Reef, Yucatán, Arc 4, 2 m, 22-08-2016, leg. L. Palomino-Alvarez; CAGoM-0447, Arcas Cay Reef, Yucatán, Arc 7, 4 m, 25-08-2016, leg. L. Palomino-Alvarez.

########## Remarks.

Specimens were found in a single location under rocks, together with *A.panamensis* and *Polycarpacartilaginea*.

########## Global distribution.

United States (Van Name 1921, 1930, 1945), Curaçao (Van Name 1924), Guadeloupe (Monniot 1983), Martinique (Monniot 2018), Mozambique (Monniot 1997), Japan (Tokioka and Nishikawa 1975), New Caledonia (Monniot 1987, 1991), Micronesia (Nishikawa 1984; Lambert 2003), and French Polynesia (Monniot and Monniot 1987a).

####### Family Perophoridae Giard, 1872

######## Genus *Ecteinascidia* Herdman, 1880

######### Ecteinascidia styeloides

Taxon classificationAnimaliaPhlebobranchiaPerophoridae

(Traustedt, 1882)

Fig. 2C

########## Material examined.

CAGoM-0441, Arc 9, 9 m, 27-08-2016, leg. L. Palomino-Alvarez; CAGoM-0442, CAGoM-0444, Bajo 10 Reef, Yucatán, B10, 7 m, 19-10-2016, leg. L. Palomino-Alvarez.

########## Remarks.

Colonies of many individuals and many sizes were found on seaweed and under rocks.

########## Global distribution.

Jamaica (Goodbody 1984, Goodbody and Cole 2006), Guadeloupe (Monniot 1983a), Belize (Goodbody 2004; Goodbody and Cole 2006), Panama (Collin et al. 2005; Rocha et al. 2005), Venezuela (Goodbody 2004; Rocha et al. 2010), Mozambique (Monniot 1997).

######### Ecteinascidia turbinata

Taxon classificationAnimaliaPhlebobranchiaPerophoridae

Herdman, 1880

########## Material examined.

CAGoM-0020, Arn 2, 7 m, 03-19-2015, leg. L. Palomino-Alvarez; CAGoM-0026, CAGoM-0027, CAGoM-0028, CAGoM-0031, CAGoM-0033, CAGoM-0034, CAGoM-0035, Chel 2, 1 m, 11-05-2015, leg. L. Palomino-Alvarez; CAGoM-0054, Pro 1, 3 m, 26-05-2015, leg. L. Palomino-Alvarez; CAGoM-0063, Chp 1, 4 m, 26-05-2015, leg. L. Palomino-Alvarez; CAGoM-0171, Arc 2, 9.4 m, 30-10-2015, leg. L. Palomino-Alvarez.

########## Remarks.

Colonies with the largest number of zooids were found in coastal lagoons on wooden piers or mangrove prop roots as well as coral reefs far from the shore. This was the most common species in Chelém (an enclosed, very salty, lagoon), comprising two morphotypes. Some had orange zooids and others had transparent, uncolored, zooids with a ring of orange along the siphon rim. This species was never found in disturbed port areas.

########## Global distribution.

United States (Van Name 1921, 1945; Plough 1978), Bermudas (Herdman 1882; Verril 1900; Berrill 1932, 1935; Monniot 1972; Van Name 1902, 1945), Cuba (Hernández-Zanuy and Carballo 2001), Jamaica (Goodbody 2003; Goodbody and Cole 2006), Turks and Caicos Islands (Millar 1962), Mexico, Yucatán Peninsula (Carballo 2000), Belize (Goodbody 2000), Panama (Collin et al. 2005), Curacao (Goodbody 1984), Venezuela (Goodbody 1984a; Rocha et al. 2010; Carballo-Pérez and Díaz 2011), Guadeloupe (Monniot 1983a), Martinique (Monniot 2018a), Guyana (Millar 1978), French Guiana (Monniot 2016), Senegal (Pérès 1949, 1951; Lafargue and Wahl 1990; Monniot and Monniot 1994), Sierra Leone (Millar 1956), Gibraltar (Naranjo and García-Gómez 1994), Baleares Islands (Ramos et al. 1993; Spain (Casso et al. 2018), France (Harant 1927, Harant and Vernières 1933, Thessalou-Legaki et al. 2012), Tunisia (Pérès 1954), and Egypt (Harant 1927).

**Figure 2.**
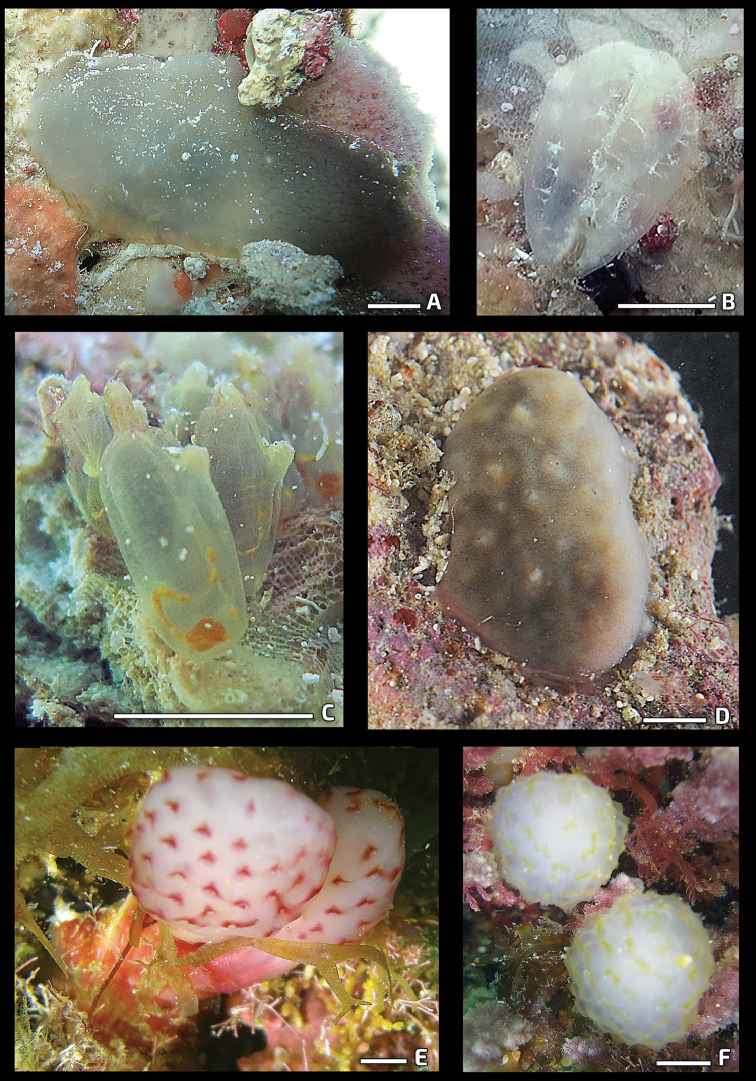
Photos of live specimens in situ in the field. **A***Ascidiapanamensis***B***Corellaminuta***C***Ecteinascidiastyeloides***D***Cystodytesroseolus***E, F**Eudistomaaff.amanitum. Scale bar: 1 cm.

###### Order Aplousobranchia Lahille, 1886

####### Family Clavelinidae Forbes & Hanley, 1848

######## Genus *Clavelina* Savigny, 1816

######### Clavelina oblonga

Taxon classificationAnimaliaAplousobranchiaClavelinidae

Herdman, 1880

########## Material examined.

CAGoM-0081, CAGoM-0082, Sey 3, 11 m, 19-03-2015, leg. L. Palomino-Alvarez; CAGoM-0093, Mad 2, 7 m, 27-05-2015, leg. L. Palomino-Alvarez.

########## Remarks.

Specimens from Seybaplaya, Campeche were associated with the hydroid *Macrorhynchiaphilippina* Kirchenpauer, 1872 (Cnidaria: Hydrozoa), from which they may gain protection from predators. The details of this association should be investigated.

########## Global distribution.

United States (Van Name 1945; Plough 1978; Lambert et al. 2005), Bermudas (Herdman 1880, 1882; Monniot 1972; Van Name 1902, 1945; Verrill 1900; Berrill 1932), Jamaica (Goodbody 1993, 2003), Southwestern Gulf of Mexico (Van Name 1921), Curaçao (Goodbody 1984; Millar 1962),Venezuela (Rocha et al. 2010), Guadeloupe (Monniot 1983), Tobago (Cole 2012), Brazil (Millar 1958; Rocha et al. 2005a; Rocha and Costa 2005; Rocha and Kremer 2005; Rodrigues et al. 1998), Azores (Monniot and Monniot 1994), Madeira (Harant 1929); Senegal (Pérès 1951; Monniot 1969; Lafargue and Wahl 1987), Spain and Italy (Ordóñez et al. 2016; Casso et al. 2018).

######### Clavelina

Taxon classificationAnimaliaAplousobranchiaClavelinidae

sp.

########## Material examined.

CAGoM-0006, CAGoM-0007, Arn 1, 2 m, 19-03-2015, leg. L. Palomino-Alvarez; CAGoM-0021, Arn 2, 6 m, 19-03-2015, leg. L. Palomino-Alvarez.

########## Remarks.

This species is dark blue with characteristics that do not match any known species and will be described elsewhere. The single colony found was small with few zooids (Table 1).

####### Family Polycitoridae Michaelsen, 1904

######## Genus *Cystodytes* Drasche, 1884

######### Cystodytes dellechiajei

Taxon classificationAnimaliaAplousobranchiaPolycitoridae

(Della Valle, 1877)

########## Material examined.

CAGoM-0135, CAGoM-0449, B10, 11 m, 17-06-2015, *leg.* L. Palomino-Alvarez.

########## Remarks.

This is the only known west Atlantic species of the genus which has been reported from nine countries, both in tropical and subtropical regions (Rocha et al. 2005, 2012). The species is very rare in the southern Gulf of Mexico (Table 1); it has been found in shallow waters of the northern Gulf of Mexico in Florida (Van Name 1945).

########## Global distribution.

United States (Van Name 1945; Plough 1978); Bermudas (Monniot 1972; Van Name 1902, 1945), Panamá (Collin et al. 2005), Les Saints, Martinique (Gravier 1955), Guyana (Millar 1977), Brazil (Millar 1977; Rocha et al. 2005), Azores (Michaelsen 1923; Monniot 1971; Monniot and Monniot 1994; Monniot 1975), Canary Islands (Ríos 1985), Senegal (Michaelsen 1915; Monniot 1969; Pérès 1949, 1951; Lafargue and Wahl 1987), Iberic Mediterranean (López-Legentil and Turon 2005), France (Harant 1927; Harant and Vernières 1933; Lafargue 1970), Italy (Drasche 1883; Brunetti 1994), Philippines (Van Name 1918), and Australia (Michaelsen 1930; Millar 1953; Kott 1990).

######### Cystodytes roseolus

Taxon classificationAnimaliaAplousobranchiaPolycitoridae

Hartmeyer, 1912

Fig. 2D

########## Material examined.

CAGoM-0114, B10, 11 m, 17-06-2015, leg. L. Palomino-Alvarez; CAGoM-0043, Pro 2, 7 m, 26-05-2015, leg. L. Palomino-Alvarez; CAGoM-0064, Chp 1, 2 m, 26-05-2015, leg. L. Palomino-Alvarez; CAGoM-0105, Mad 4, 5 m, 17-06-2015, leg. L. Palomino-Alvarez; CAGoM-0465, B10, 11 m, 17-06-2015, leg. L. Palomino-Alvarez.

########## Remarks.

*Cystodytesroseolus* might have been found in Atlantic Panama in 2003 but identification needs to be confirmed due to the disjunct distribution (Rocha et al. 2005). This second report of the species in the Gulf of Mexico indicates that this species occurs on both sides of the Atlantic. Finding this species in Progreso Harbor and nearby locations suggests that this species was introduced to the Yucatán peninsula from Africa by ship transport.

########## Global distribution.

Senegal (Pérès 1949; Monniot 1969; Lafargue and Wahl 1987; Monniot and Monniot 1994), South Africa (Hartmeyer 1912; Michaelsen 1919, 1934; Millar 1962), Seychelles (Michaelsen 1919).

######## Genus *Eudistoma* Caullery, 1909

######### Eudistoma aff. amanitum

Taxon classificationAnimaliaAplousobranchiaPolycitoridae

Paiva & Rocha, 2018

Fig. 2E, F

########## Material examined.

CAGoM-0074, Mad 2, 9 m, 27-05-2015, leg. L. Palomino-Alvarez; CAGoM-0070, Mad 2, 10 m, 27-05-2015, leg. L. Palomino-Alvarez; CAGoM-0100, Mad 4, 12 m, 17-06-2015, leg. L. Palomino-Alvarez; CAGoM-0112, B10, 7 m, 17-06-2015, leg. L. Palomino-Alvarez; CAGoM-0115, B10, 9 m, 17-06-2015, leg. L. Palomino-Alvarez; CAGoM-0139, CAGoM-0140, CAGoM-0142, Lar 1, 10 m, 07-10-2015, leg. L. Palomino- Alvarez; CAGoM-0149, CAGoM-0150, Lar 2, 10 m, 07-10-2015, leg. L. Palomino-Alvarez; CAGoM-0152, Lar 2, 12 m, 07-10-2015, leg. L. Palomino-Alvarez; CAGoM-0163, Lar 3, 12 m, 07-10-2015, leg. L. Palomino-Alvarez.

########## Remarks.

Colonies from Mexico and Panama vary by location in the number of heads per peduncle and shape, zooid size, zooid position within the tunic, and color (Paiva and Rocha 2018). Gonads were undeveloped and no larvae were found, thus this species identification remains to be confirmed.

########## Global distribution.

Southern Gulf of Mexico (described herein) and Panama (Paiva and Rocha 2018).

######### Eudistoma clarum

Taxon classificationAnimaliaAplousobranchiaPolycitoridae

(Van Name, 1902)

########## Material examined.

CAGoM-0041, Pro 3, 8 m, 26-05-2015, leg. L. Palomino-Alvarez; CAGoM-0051, Pro 1, 14 m, 26-05-2015, leg. L. Palomino-Alvarez; CAGoM-0077, Mad 3, 12 m, 27-05-2015, leg. L. Palomino-Alvarez; CAGoM-0103, Mad 4, 9 m, 27-05-2015, leg. L. Palomino-Alvarez; CAGoM-00166, Lar 3, 12 m, 07-10-2015, leg. L. Palomino-Alvarez.

########## Remarks.

Records of *E.clarum* have been found in mangroves and to a depth of 20 m in coral reefs (Goodbody, 2000). We found specimens mainly in coral reefs and near shore in places with strong anthropogenic impact.

########## Global distribution.

United States (Van Name 1921), Bermudas (Van Name 1902, 1945; Berrill 1932; Monniot 1972), Belize (Goodbody 2000), Panama (Collin et al. 2005; Rocha et al. 2005), Bonaire (Millar 1962), Venezuela and Caribbean Islands (Millar 1962; Goodbody 1984), Tobago (Cole 2012), Guadeloupe (Monniot 1983), Senegal (Pérès 1949), and French Polynesia (Monniot and Monniot 1987a).

######### Eudistoma hepaticum

Taxon classificationAnimaliaAplousobranchiaPolycitoridae

(Van Name, 1921)

Fig. 3G

########## Material examined.

CAGoM-0052, Pro 1, 6 m, 26-05-2015, leg. L. Palomino-Alvarez; CAGoM-0068, Chp1, 26-05-2015, 2 m, leg. L. Palomino-Alvarez; CAGoM-0091, Sey 2, 12-06-2015, 11 m, leg. Palomino-Palomino Alvarez; CAGoM-0088, Sey 1, 12-06-2015, 11 m, leg. Palomino-Palomino Alvarez; CAGoM-0107, Mad 4, 17-06-2015, 9 m, leg. L. Palomino-Alvarez; CAGoM-0072, Mad 2, 13 m, 27-05-2015, leg. L. Palomino-Alvarez; CAGoM-0039, Cel 1, 11-05-2015, 1 m, leg. L. Palomino-Alvarez, CAGoM-0116, B10, 9 m, 17-06-2015, leg. L. Palomino-Alvarez; CAGoM-0087, Sey 2, 11 m, 12-06-2015, leg. L. Palomino-Alvarez.

########## Remarks.

We found large (about 20 cm in diameter) purple or blue colonies on cement columns in Progreso Harbor and smaller colonies were found on coral reefs. This is the most common species of *Eudistoma* in the region.

########## Global distribution.

United States (Van Name 1945; Plough 1978), Bermudas (Van Name 1902), Jamaica (Goodbody 2003, Van Name 1921), St. Thomas (Van Name 1921), Mexico (Van Name 1945), Curaçao (Goodbody 1984b), Venezuela (Goodbody 1984a), and Guadeloupe (Monniot 1983c).

######### Eudistoma obscuratum

Taxon classificationAnimaliaAplousobranchiaPolycitoridae

(Van Name, 1902)

########## Material examined.

CAGoM-0073, Mad 2, 8 m, 27-05-2015, leg. L. Palomino-Alvarez; CAGoM-0101, CAGoM-0109, Mad 4, 12 m, 17-06-2015, leg. L. Palomino-Alvarez; CAGoM-0159, Lar 2, 14 m, 07-10-2015, leg. L. Palomino-Alvarez.

########## Remarks.

Colonies are small and found beneath rocks and on bivalve shells.

########## Global distribution.

United States (Van Name 1921, 1945), Bermuda (Monniot 1972; Van Name 1902, 1945), Virgin Islands (Van Name 1921, 1945), and Belize (Goodbody 2000).

######### Eudistoma olivaceum

Taxon classificationAnimaliaAplousobranchiaPolycitoridae

(Van Name, 1902)

Fig. 3H

########## Material examined.

CAGoM-0016, Mha, 1 m, 12-03-2015, leg. L. Palomino-Alvarez; CAGoM-0078, Chp 2, 0.5 m, 11-06-2015, leg. L. Palomino-Alvarez; CAGoM-0025, Mad 4, 9 m, 04-04-2015, leg. L. Palomino-Alvarez; CAGoM-0060, Pro 1, 8 m, 26-05-2015, leg. L. Palomino-Alvarez; CAGoM-0036, Chel 2, 11-05-2015, 1 m, leg. L. Palomino-Alvarez.

########## Remarks.

Zooids of some samples have a third opening at the base of the thorax through which fecal pellets are expelled. While colonies appeared healthy, this third opening may have been due to body wall rupture caused by obstruction of the atrial canal by incubating larvae in the atrial cavity or excess sediments in the water. The appearance of third siphons may be induced by experimental injuries in a few solitary ascidians (Jeffery et al. 2015).

########## Global distribution.

United States (Van Name 1921, 1945; Plough 1978), Bermudas (Berrill 1932; Monniot 1972; Van Name 1902, 1945), Jamaica (Goodbody 2003), Cuba (Van Name 1921), Puerto Rico (Van Name 1921), Guadeloupe (Monniot 1983c), Belize (Goodbody 2004), Curaçao (Van Name 1924; Millar 1962; Goodbody 1984), Venezuela (Millar 1962; Goodbody 1984a), Tobago (Cole 2012), Senegal (Lafargue and Wahl 1987), Micronesia (Nishikawa 1984).

######### Eudistoma recifense

Taxon classificationAnimaliaAplousobranchiaPolycitoridae

Millar, 1977

Fig. 3I

########## Material examined.

CAGoM-0071, Mad 2, 11 m, 27-05-2015, leg. L. Palomino-Alvarez; CAGoM-0047, Pro 1, 8 m, 26-05-2015, leg. L. Palomino-Alvarez; CAGoM-0137, B10, 11 m, 17-06-2015, leg. L. Palomino-Alvarez.

########## Remarks.

Commonly found on grass beds, coral reefs and cement columns of harbors near the shore.

########## Global distribution.

Southern Gulf of Mexico (present study) and Brazil (Millar 1977; Oliveira et al. 2014).

**Figure 3.**
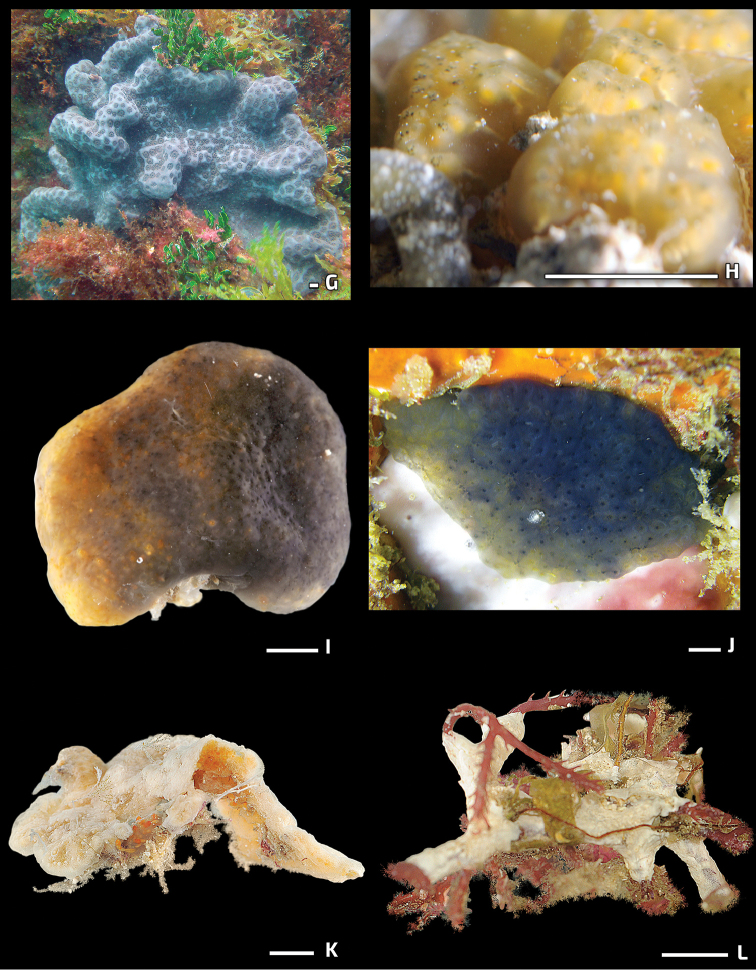
Photos of live specimens in the field (continued). **G***Eudistomahepaticum***H***Eudistomaolivaceum***I***Eudistomarecifense***J***Distapliabermudensis***K***Polysyncratonamethysteum* (preserved specimen) **L***Lissoclinumfragile*. Scale bar: 1 cm.

####### Family Stomozoidae Kott, 1990

######## Genus *Stomozoa* Kott, 1957

######### Stomozoa roseola

Taxon classificationAnimaliaAplousobranchiaStomozoidae

(Millar, 1955)

########## Material examined.

CAGoM-0076, Mad 3, 12 m, 27-05-2015, leg. L. Palomino-Alvarez; CAGOM-69, Chp 1, 5 m, 26-05-2015, leg. L. Palomino-Alvarez.

########## Remarks.

Colonies were found on dead coral and between large rocks. The tunic is very firm and dark purple, similar to colonies from the Red Sea and Madagascar.

########## Global distribution.

United States (Van Name 1945; Plough 1978); Mexico (Van Name 1945), French Guiana (Monniot 2016), Brazil (Millar 1977), South Africa (Millar 1955), Madagascar (Monniot 2012), Red Sea (Kott 1957), Indonesia (Monniot and Monniot 1996), and New Caledonia (Monniot 1988).

####### Family Holozoidae Berrill, 1950

######## Genus *Distaplia* Della Valle, 1881

######### Distaplia bermudensis

Taxon classificationAnimaliaAplousobranchiaHolozoidae

Van Name, 1902

Fig. 3J

########## Material examined.

CAGoM-00102, Mad 4, 14 m, 17-06-2015, leg. L. Palomino-Alvarez; CAGoM-0095 Mad 4, 17-06-2015, 9 m, leg. L. Palomino-Alvarez.

########## Remarks.

Although this species is common in many Caribbean countries, we found it in only one location.

########## Global distribution.

United States (Van Name 1921, 1945; Plough 1978), Bermudas (Van Name 1902, 1945; Berrill 1932; Gravier 1955; Monniot 1972), Cuba (Hernandez 1990), Jamaica (Goodbody 2003), Puerto Rico (Van Name 1921), Virgin Islands, St Thomas (Van Name 1921), Guadeloupe (Monniot 1983); Belize (Goodbody 2004), Panamá (Collin et al. 2005, Rocha et al. 2005), Curaçao (Millar 1962; Goodbody 1984), Venezuela (Millar 1962; Goodbody 1984; Rocha et al. 2010), Guyana (Millar 1978), French Guiana (Monniot 2016), Brazil (Millar 1958, 1977; Rodrigues and Rocha 1993; Rodrigues et al. 1998; Rocha et al. 2005; Rocha and Costa 2005; Rocha and Kremer 2005), Senegal (Pérès 1949), and Italy (Mastrototaro and Brunetti 2006).

####### Family Didemnidae Giard, 1872

######## Genus *Polysyncraton* Nott, 1892

######### Polysyncraton amethysteum

Taxon classificationAnimaliaAplousobranchiaDidemnidae

Van Name, 1902

Fig. 3K

########## Material examined.

CAGoM-0158, Lar 2, 9 m, 07-10-2015, leg. L. Palomino-Alvarez; CAGoM-0118, B10, 9 m, 17-06-2015, leg. L. Palomino-Alvarez.

########## Remarks.

Colony found on corals and rocks. Orange zooids when alive and in preservation.

########## Global distribution.

United States (Van Name 1921), Bermuda (Van Name 1902, 1921, 1945), Puerto Rico (Van Name 1945), Guadeloupe (Gravier 1955), Martinique (Gravier 1955); Colombia (Van Name 1945), French Guiana (Monniot 2016), Brazil (Millar 1958, 1977; Rodrigues and Rocha 1993; Rocha et al. 2005; Rocha and Kremer 2005), Cape Verde (Monniot and Monniot 1967), Senegal (Pérès 1948, 1949), Ghana (Millar 1953), Tunisia (Pérès 1954).

######## Genus *Lissoclinum* Verrill, 1871

######### Lissoclinum fragile

Taxon classificationAnimaliaAplousobranchiaDidemnidae

(Van Name, 1902)

Fig. 3L

########## Material examined.

CAGoM-0143, Lar 1, 12 m, 07-10-2015, leg. L. Palomino-Alvarez.

########## Remarks.

We found colonies in a single location growing on algae. *Lissoclinumfragile* is reported from tropical and subtropical regions where it is very common in marinas on artificial substrates, which suggests that is has been extensively introduced while the original geographical distribution remains unknown.

########## Global distribution.

United States (Van Name 1921; Lambert et al. 2005), Bermuda (Van Name 1902; Berrill 1932), St Thomas (Van Name 1921), Jamaica (Goodbody 1993), Guadeloupe (Monniot 1983a), Belize (Goodbody 2004), Costa Rica (Tokioka 1972), Curaçao (Millar 1962; Van Name 1924), Venezuela (Millar 1962; Rocha et al. 2010), Tobago (Cole 2012), Brazil (Rodrigues et al. 1998; Rocha and Kremer 2005; Rocha and Faria 2005), Azores (Monniot 1974), Sierra Leone (Monniot and Monniot 1994), Madagascar (Vasseur 1970), Persian Gulf (Monniot 1997), India (Renganathan 1982), Philippines (Tokioka 1967), Indonesia (Lafargue and Vasseur 1989), Japan (Tokioka 1954), New Caledonia (Monniot 1992), Guam (Monniot and Monniot 2001; Lambert 2003), and French Polynesia (Monniot and Monniot 1987a; Monniot et al. 1985).

######## Genus *Didemnum* Savigny, 1816

######### Didemnum duplicatum

Taxon classificationAnimaliaAplousobranchiaDidemnidae

Monniot, 1983

########## Material examined.

CAGoM-050, CAGoM-0475, Pro 1, 13 m, 26-05-2015, leg. L. Palomino-Alvarez; CAGoM-0080, Sey 3, 8 m, 12-06-2015, leg. L. Palomino-Alvarez; CAGoM-0108, Mad 4, 11 m, 17-06-2015, leg. L. Palomino-Alvarez; CAGoM-0126, CAGoM-0133, B10, 7 m, 17-06-2015, leg. L. Palomino-Alvarez; CAGoM-0186, Arc 6, 8 m, 30-10-2015, leg. L. Palomino-Alvarez.

########## Remarks.

Colonies were found only near shore (harbors) and on artificial reefs. Recent molecular unpublished data (RMR) suggests that this might by a complex of three species.

########## Global distribution.

United States (Lambert et al. 2005), Jamaica (Goodbody 2003), Guadeloupe (Monniot 1983a), Belize (Goodbody 2000), Curaçao (Goodbody 1984), Venezuela (Goodbody 1984; Rocha et al. 2010), Tobago (Cole 2012), French Guiana (Monniot 2016).

######### Didemnum granulatum

Taxon classificationAnimaliaAplousobranchiaDidemnidae

Tokioka, 1954

########## Material examined.

CAGoM-0075, Mad 3, 7 m, 27-05-2015, leg. L. Palomino-Alvarez; CAGoM-0153, Lar 2, 10 m, 07-10-2015, leg. L. Palomino-Alvarez.

########## Remarks.

With a global distribution, *D.granulatum* is known to rapidly colonize artificial substrates (Oren and Benayahu 1998), so it may be widely introduced, but its origin is unknown. In the southern Gulf of Mexico colonies were found on corals, rocks and algae near shore.

########## Global distribution.

Panama (Rocha et al. 2005), French Guiana (Monniot 2016), Brazil (Dias et al. 2012; Paiva et al. 2015), Senegal (Monniot and Monniot 1994), South Africa (Monniot et al. 2001), Red Sea (Shenkar 2012), Hong Kong (Kott and Goodbody 1982), Philippines (Monniot and Monniot 2001), Papua New Guinea (Monniot and Monniot 2001), Japan (Tokioka 1954), Australia (Kott 2001), New Caledonia (Monniot 1995), French Polynesia (Monniot and Monniot 1987a), and Fiji (Kott 1981).

####### Family Polyclinidae Milne Edwards, 1841

######## Genus *Polyclinum* Savigny, 1816

######### Polyclinum constellatum

Taxon classificationAnimaliaAplousobranchiaPolyclinidae

Savigny, 1816

Fig. 4M

########## Material examined.

CAGoM-0731, CAGoM-0732, CAGoM-0736, Chel 1, 0 m, 21-03-2018, leg. R.M. Rocha.

########## Remarks.

This is another widespread species that was probably introduced in the southern Gulf of Mexico, yet we only found it in one harbor. The high salinity tolerance has been observed in Margarita Island, Venezuela where the species has also been found in an estuary with salinity > 50 ppt (Rocha et al. 2010).

########## Global distribution.

United States (Van Name 1945), Gulf of Mexico (Van Name 1945; Lambert et al. 2005), Bahamas (Van Name 1945), Bermuda (Monniot 1972), Cuba (Van Name 1945), Jamaica (Van Name 1945, Goodbody 1993), Puerto Rico (Van Name 1921), Guadeloupe (Monniot 1983b), Martinique (Gravier 1955), Belize (Goodbody 2000), Panama (Carman et al. 2010), Colombia (Van Name 1945), Curaçao (Millar 1962; Goodbody 1984), Venezuela (Rocha et al. 2010; Carballo-Pérez and Díaz 2011), French Guiana (Monniot 2016), Brazil (Millar 1958; Rodrigues and Rocha 1993; Rocha et al. 2005, 2011), South Africa (Millar 1955), Mozambique (Michaelsen 1919, Monniot and Monniot 1976), Madagascar (Vasseur 1970), Mauritius Island (Savigny 1816), Persian Gulf (Monniot and Monniot 1997), Japan (Tokioka 1963, 1967), China (Michaelsen 1923), New Caledonia (Monniot 2007).

####### Family Euherdmaniidae Ritter, 1904

######## Genus *Euherdmania* Ritter, 1904

######### Euherdmania fasciculata

Taxon classificationAnimaliaAplousobranchiaEuherdmaniidae

Monniot, 1983

########## Material examined.

CAGoM-00471, Pro 1, 13 m, 26-05-2015, leg. L. Palomino-Alvarez.

########## Remarks.

The specimen was found in a disturbed environment (near-shore artificial reef).

########## Global distribution.

Southern Gulf of Mexico (present study), Guadeloupe (Monniot 1983b), French Guiana (Monniot 2016)

######### Euherdmania aff. vitrea

Taxon classificationAnimaliaAplousobranchiaEuherdmaniidae

Millar, 1961

Fig. 4N

########## Material examined.

CAGoM-00104, Mad 4, 11 m, 17-06-2015, leg. L. Palomino-Alvarez; CAGoM-00120, B10, 10 m, 17-06-2015, leg. L. Palomino-Alvarez.

########## Remarks.

Morphological patterns of colony shape, zooids completely embedded in the tunic, conspicuous musculature throughout the body, number of siphon lobes, number of stigmatal rows, and the testis position correspond with *E.vitrea* by Millar (1961), but the absence of the developed gonads and larvae prevent confirmation of this species. Also, the tunic is dark red colored while all colonies collected in Brazil are uncolored.

########## Global distribution.

Southern Gulf of Mexico (present study), Brazil (Millar 1961; Rocha et al. 2005).

**Figure 4.**
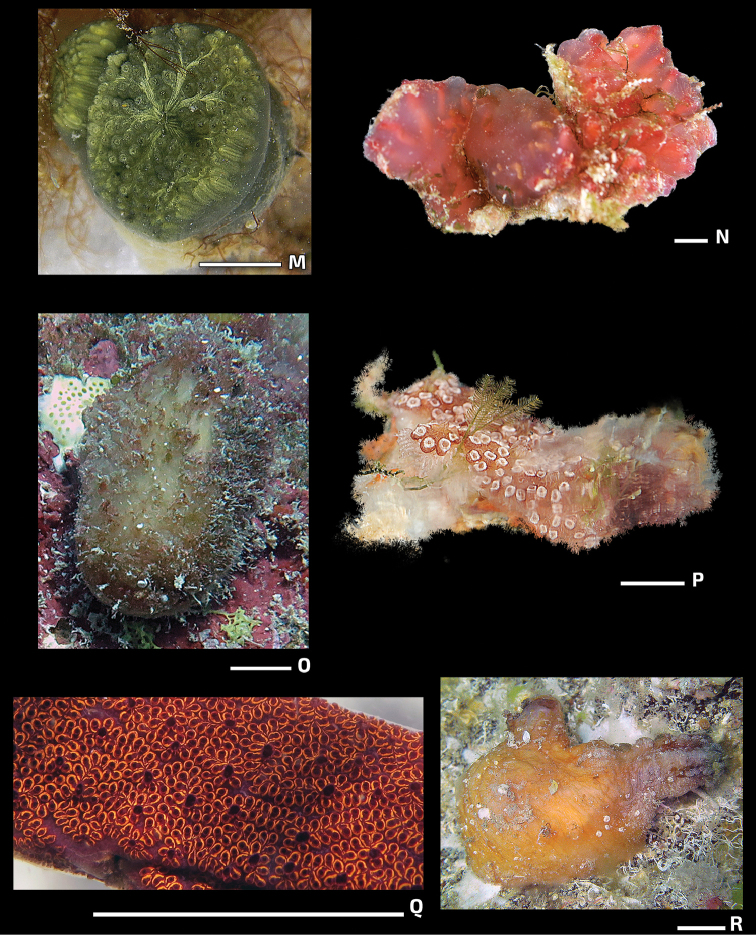
Photos of live specimens in the field (continued). **M***Polyclinumconstellatum***N**Euherdmaniaaff.vitrea**O***Polycarpacartilaginea***P***Botrylloidesmagnicoecus***Q***Botrylloidesniger***R***Microcosmusexasperatus*. Scale bar: 1 cm.

###### Order Stolidobranchia Lahille, 1886

####### Family Styelidae Sluiter, 1895

######## Genus *Polycarpa* Heller, 1877

######### Polycarpa cartilaginea

Taxon classificationAnimaliaStolidobranchiaStyelidae

(Sluiter, 1898)

Fig. 4O

########## Material examined.

CAGoM-0010, Arn 1, 12 m, 19-03- 2015, leg. L. Palomino-Alvarez; CAGoM-0176, Arc 1, 4 m, 19-03- 2015, leg. L. Palomino-Alvarez; CAGoM-0364, CAGoM-0365, Arc 11, 7 m, 20-08- 2016, leg. L. Palomino-Alvarez; CAGoM-0386, CAGoM-00468, Arc 4, 6 m, 22-08-2016, leg. L. Palomino-Alvarez; CAGoM-0408, CAGoM-412, CAGoM-0420, CAGoM-0421, Arc 12, 6 m, 24-08- 2016, leg. L. Palomino-Alvarez; CAGoM-0426, CAGoM-0479, Arc 7, 9 m, 25-08-2016, leg. L. Palomino-Alvarez; CAGoM-0437, Acr 10, 12 m, 26-08- 2016, leg. L. Palomino-Alvarez.

########## Remarks.

All the specimens from Arcas Cay Reef were found under large rocks with other species of ascidians (*Ascidiapanamensis*, *Corellaminuta* and *Ecteinascidiastyeloides*).

########## Global distribution.

Belize (Goodbody 2000), Panama (Collin et al. 2005; Rocha et al. 2005), Curaçao (Millar 1962; Van der Sloot 1969; Goodbody 1984), Colombia (Sluiter 1898), Guadeloupe (Monniot 1983), Martinique (Monniot 2018b).

######### Polycarpa spongiabilis

Taxon classificationAnimaliaStolidobranchiaStyelidae

Traustedt, 1883

########## Material examined.

CAGoM-0022, Arn 2, 6 m, 19-03- 2015, leg. L. Palomino-Alvarez; CAGoM-0448, Arc 10, 4 m, 26-08-2016, leg. L. Palomino-Alvarez.

########## Remarks.

Amphipods were found between folds of the pharynx of *P.spongiabilis* (two males of *Leucothoewuriti* Thomas & Klebba, 2007).

########## Global distribution.

United States (Van Name 1921, 1945; Plough 1978), Bermuda (Verrill 1900; Van Name 1902, 1945; Gravier 1955; Monniot 1972), Cuba (Hernandez 1990), Jamaica (Sluiter 1898; Goodbody 1993), Puerto Rico (Van Name 1921, 1930), St. Thomas (Traustedt 1883), Guadeloupe (Gravier 1955; Monniot 1983b), Martinique (Monniot 2018b), Belize (Goodbody 2000), Panamá (Collin et al. 2005; Rocha et al. 2005), Curaçao (Sluiter 1898; Goodbody 1984), Venezuela (Sluiter 1898; Goodbody 1984; Rocha et al. 2010), Tobago (Cole 2012), Guyana (Millar 1978), Brazil (Rodrigues 1962; Millar 1977; Rocha and Kremer 2005).

######## Genus *Botrylloides* Milne Edwards, 1841

######### Botrylloides magnicoecus

Taxon classificationAnimaliaStolidobranchiaStyelidae

(Hartmeyer, 1912)

Fig. 4P

########## Material examined.

CAGoM-0125, B10, 11 m, 17-06-2015, leg. L. Palomino-Alvarez.

########## Remarks.

This is the first record of *B.magnicoecus* in the Gulf of Mexico. General characteristics agree with descriptions by Brunetti (2010), but caecum size in the Mexico specimens is larger.

########## Global distribution.

According to Brunetti (2010) the records from South Africa have characteristics in agreement with the type and are trustful (Michaelsen 1934; Millar 1955). Recently a new record from French Guiana (Monniot 2016) has been published. A more detailed study of the species in the type locality should be performed to permit the revision of other records in the Atlantic (Guadeloupe – Monniot 1983b, Belize – Goodbody 2004).

######### Botrylloides niger

Taxon classificationAnimaliaStolidobranchiaStyelidae

Herdman, 1886

Fig. 4Q

########## Material examined.

CAGoM-0029, CAGoM-0030, CAGoM-0032, CAGoM-0037, CAGoM-0038, Chel 2, 1 m, 11-05-2015, leg. L. Palomino-Alvarez; CAGoM-0040, Cel 2, 1 m, 11-05-2015, leg. L. Palomino-Alvarez; CAGoM-0185, Arc 3, 3 m, 30-10-2015, leg. L. Palomino-Alvarez.

**Photographed record (no specimens in collection)**: VeR, 8 m, 20-09-2015, L. Palomino-Alvarez

########## Remarks.

*Botrylloidesniger* is among the species considered common and abundant in tropical waters (Rocha et al. 2010) with a widespread geographical distribution, where it attaches to natural and artificial substrates (Sheets et al. 2016 – as *B.nigrum*). We found it beneath rocks, on coral reefs, in ports and lagoons.

########## Global distribution.

United States (Van Name 1945), Mexico (Van Name 1945), Bermuda (Herdman 1886; Van Name 1902, 1945; Monniot 1972), Belize (Goodbody 2000), Panama (Carman et al. 2011), Curaçao (Goodbody 1984), Bonaire (Millar 1962), Venezuela (Rocha et al. 2010; Carballo-Pérez and Díaz 2011), Cuba (Van Name 1945), Jamaica (Goodbody 2003), Puerto Rico (Van Name), Guadeloupe (Monniot 1983b), Martinique (Monniot 2018b as *B.nigrum*), Antilles (Gravier 1955; Van Name 1930), Tobago (Cole 2012), Brazil (Rodrigues 1962; Rocha and Costa 2005; Rocha and Kremer 2005; Rocha and Bonnet 2009; Dias et al. 2012), Morocco (Millar 1698), Senegal (Monniot 1969), Israel Mediterranean (Sheets et al. 2016), Singapore (Sheets et al. 2016), Somalia (Millar 1988), Madagascar (Vasseur 1970), French Polynesia (Monniot et al. 1985).

####### Family Pyuridae Hartmeyer, 1908

######## Genus *Pyura* Molina, 1782

######### Pyura

Taxon classificationAnimaliaStolidobranchiaPyuridae

sp.

########## Material examined.

CAGoM-0129, CAGoM-0128, CAGoM-0134, B10, 11 m, 17-06-2017, leg. L. Palomino-Alvarez; CAGoM-0160, CAGoM-0162, CAGoM-0146, Pro 1, 7 m, 26-05-2015, leg. L. Palomino-Alvarez.

########## Remarks.

Specimens were compared with Panamanian specimens and are similar to a new species being described (Skinner et al. in press). In Yucatan peninsula they are 9–10 cm long, and were found mainly on coral reefs. Oral tentacles are more numerous (43–58) and one of the specimens had the following vessel formula:

E 11 (28) 9 (28) 6 (34) 6 (36) 5 (33) 5 (31) 6 LD 3 (30) 5 (28) 5 (27) 6 (32) 6 (30) 6 (22) 10. All other characters were within the variation found in Panama.

######## Genus *Microcosmus* Heller, 1877

######### Microcosmus exasperatus

Taxon classificationAnimaliaStolidobranchiaPyuridae

Heller, 1878

Fig. 4R

########## Material examined.

CAGoM-0131, B10, 11 m, 17-06-2015, leg. L. Palomino-Alvarez; CAGoM-0048, Pro 1, 8 m, 26-05-2015, leg. L. Palomino-Alvarez; CAGoM-00476, Chp 1, 5 m, 26-05-2015, leg. L. Palomino-Alvarez; CAGoM-0731, Sis, 1 m, 21-03-2018, leg. L. Palomino-Alvarez.

########## Remarks.

Can be very common on mangrove prop roots in the Caribbean Sea (Panama – Rocha el al. 2005, Venezuela – Rocha et al. 2010). Here we found it mainly in harbors, with > 20 specimens found in Sisal harbour, suggesting that it was introduced.

########## Global distribution.

United States (Van Name 1921, 1945), Bermudas (Berrill 1932; Monniot 1972), Jamaica (Heller 1878; Goodbody 2003), Belize (Goodbody 2000), Panamá (Collin et al. 2005; Carman et al. 2011), Curaçao (Van Name 1924; Millar 1962; Goodbody 1984), Venezuela (Rocha et al. 2010; Carballo-Pérez and Díaz 2011), Tobago (Cole 2012), Martinique (Gravier 1955; Monniot 2018c), Antilles (Sluiter 1898; Van Name 1921, 1931), Guadeloupe (Monniot 1983), Brazil (Rodrigues 1962; Rodrigues et al. 1998; Monniot and Monniot 2001; Rocha et al. 2012; Rocha and Costa 2005; Rocha and Kremer 2005; Rocha et al. 2005), Azores (Harant 1929), Cape Verde (Harant 1929), Mediterranean sea (Streftaris et al. 2005; Turon et al. 2007), Red Sea (Shenkar 2012), Philippine Sea (Van Name 1918), Mindoro (Tokioka 1970), Truuk Islands (Nishikawa 1984), Guam (Lambert 2003), Australia (Kott 1985; Monniot 1992).

####### Family Molgulidae Lacaze-Duthiers, 1877

######## Genus *Molgula* Forbes, 1848

######### Molgula occidentalis

Taxon classificationAnimaliaStolidobranchiaMolgulidae

Traustedt, 1883

########## Material examined.

CAGoM-0734, Sis, 1 m, 21-03-2018, leg. L. Palomino-Alvarez.

########## Remarks.

The single specimen was found with *M.exasperatus* on a cement column.

########## Global distribution.

Unites States (Van Name 1945), Panama (Collin et al., 2005; Rocha et al. 2005), Curaçao (Van Name 1924; Millar 1962; Goodbody 1984), Venezuela (Goodbody 1984; Rocha et al. 2010), Virgin Islands (Traustedt 1883), Puerto Rico (Van Name 1921, 1930), Brazil (Monniot 1970), Senegal (Pérès 1949; Monniot 1969; Lafargue and Wahl 1987; Monniot and Monniot 1994), Italy (Monniot 1970).

## Discussion

With this first checklist from the southern Gulf of Mexico, we list 31 species, five in the order Phlebobranchia, 19 Aplousobranchia and seven Stolidobranchia. Ascidians found in the southern Gulf of Mexico comprise 24% of the species, 24% of the genera and 60% of the families of ascidians that have been found throughout the Gulf of Mexico (Van Name 1945; Abbott 1951; Carballo 2000; Lambert et al. 2005; Cole and Lambert 2009). Also, they comprise 7% of the species, 22% of the genera and 53% of the families reported from the Atlantic Ocean (Rocha et al. 2012). Styelidae and Polycitoridae are the most species-rich families.

The number of species we found is surprisingly less than expected and that have been reported from other regions of the Caribbean, including Belize, Bocas del Toro (Panama), Jamaica and Guadeloupe, while similar to the number of species found in Cuba, Curaçao and Puerto Rico (Rocha et al., 2005). A possible explanation for fewer species is simply variation in sampling effort among studies, or that greater diversity has been found in association with mangrove roots in some of those studies. Mangroves are less common in the southern Gulf of Mexico surveyed here and therefore they were sampled less often in this study. In the present study we visited eight coral reefs, where ascidians are mainly found beneath pieces of dead coral and in crevices and pits where they are not easily found. Sampling effort was somewhat greater in the two sites with more species (Madagascar and Bajo 10). Four sites were in harbors or associated with marinas and urban construction, one of which had many species (Progreso). Harbors are known as entrances for exotic species, among which only *C.roseolus*, *P.constellatum* and *M.exasperatus* are likely to be introduced. Some species found in anthropogenic sites are widely distributed and have been introduced elsewhere (e.g., *D.perlucidum*, *L.fragile*, *E.turbinata*, *P.nigra* – Renganathan 1982; Monniot et al. 1985; Sheehy and Vik 2010; Thessalou et al. 2012; Vandepas et al. 2015).

Major affinities of the ascidian fauna in southern Gulf of Mexico are with the Caribbean Sea (25 shared species) and West Atlantic countries with tropical or warm waters (21 species), and only then with the northern region of the Gulf (19 species). Half of the species are also found in the east Atlantic region, and 13 species have wide geographical distribution including either or both Indian and Pacific oceans waters (Table 3). In contrast, we found a few species that are very common elsewhere in the Caribbean Sea, including *Rhopalaeaabdominalis*, *Ascidiacurvata*, *A.interrupta*, *Symplegmarubra*, *S.brakenhielmi*, *Pyuravittata*, and *Herdmaniapallida*. Whether there are oceanographic or biological barriers preventing species from entering the southern Gulf of Mexico remains to be tested. Also, increasing sampling effort will certainly uncover more species.

**Table 3.** World distribution of the ascidian species found in the present survey in southern Gulf of Mexico.

	Gulf N	Gulf S	Caribbean	West Atl	East Atl	Medit	Indian	Pacif
Total #	19	31	25	21	16	9	10	10
* Ascidia panamensis *		x	x					
* Phallusia nigra *	x	x	x	x	x	x	x	
* Corella minuta *	x	x	x				x	x
* Ecteinascidia styeloides *		x	x				x	
* Ecteinascidia turbinata *	x	x	x	x	x	x		
* Clavelina oblonga *	x	x	x	x	x	x		
*Clavelina* sp.		x						
* Cystodytes dellechiajei *	x	x	x	x	x	x		x
* Cystodytes roseolus *		x			x		x	
Eudistoma aff. amanitum		x	x					
* Eudistoma clarum *	x	x	x		x			x
* Eudistoma hepaticum *	x	x	x					
* Eudistoma obscuratum *	x	x	x	x				
* Eudistoma olivaceum *	x	x	x	x	x			x
* Eudistoma recifense *		x		x				
* Stomozoa roseola *	x	x		x	x		x	x
* Distaplia bermudensis *	x	x	x	x	x	x		
* Polysyncraton amethysteum *	x	x	x	x	x	x		
* Lissoclinum fragile *	x	x	x	x	x		x	x
* Didemnum duplicatum *	x	x	x	x				
* Didemnum granulatum *		x	x	x	x		x	x
* Polyclinum constellatum *	x	x	x	x	x		x	x
* Euherdmania fasciculata *		x	x	x				
Euherdmania aff. vitrea		x		x				
* Polycarpa cartilaginea *		x	x					
* Polycarpa spongiabilis *	x	x	x	x				
* Botrylloides magnicoecus *		x		x	x			
* Botrylloides niger *	x	x	x	x	x	x	x	x
*Pyura* sp.		x	x					
* Microcosmus exasperatus *	x	x	x	x	x	x	x	x
* Molgula occidentalis *	x	x	x	x		x		

## Supplementary Material

XML Treatment for Ascidia panamensis

XML Treatment for Phallusia nigra

XML Treatment for Corella minuta

XML Treatment for Ecteinascidia styeloides

XML Treatment for Ecteinascidia turbinata

XML Treatment for Clavelina oblonga

XML Treatment for Clavelina

XML Treatment for Cystodytes dellechiajei

XML Treatment for Cystodytes roseolus

XML Treatment for Eudistoma aff. amanitum

XML Treatment for Eudistoma clarum

XML Treatment for Eudistoma hepaticum

XML Treatment for Eudistoma obscuratum

XML Treatment for Eudistoma olivaceum

XML Treatment for Eudistoma recifense

XML Treatment for Stomozoa roseola

XML Treatment for Distaplia bermudensis

XML Treatment for Polysyncraton amethysteum

XML Treatment for Lissoclinum fragile

XML Treatment for Didemnum duplicatum

XML Treatment for Didemnum granulatum

XML Treatment for Polyclinum constellatum

XML Treatment for Euherdmania fasciculata

XML Treatment for Euherdmania aff. vitrea

XML Treatment for Polycarpa cartilaginea

XML Treatment for Polycarpa spongiabilis

XML Treatment for Botrylloides magnicoecus

XML Treatment for Botrylloides niger

XML Treatment for Pyura

XML Treatment for Microcosmus exasperatus

XML Treatment for Molgula occidentalis
